# The complete chloroplast genome of *Ormosia nuda* (fabaceae), an endemic species in China

**DOI:** 10.1080/23802359.2021.1911709

**Published:** 2021-06-21

**Authors:** Yin-Huan Wang

**Affiliations:** School of Primary Education, Chongqing Normal University, Chongqing, China

**Keywords:** Chloroplast genome, *Ormosia nuda*, phylogenetic analysis, Ormosia

## Abstract

*Ormosia nuda* is a legume species endemic to China. The chloroplast genome (plastome) of this species was assembled in this study. The total plastome size is 173,789 bp in length, containing a large single-copy (LSC) region of 73,847 bp, a small single-copy (SSC) region of 18,744 bp, and two inverted repeat (IR) regions of 40,599 bp which have expanded about 15 kb into LSC. The plastome encodes a total of 111 unique genes, including 77 protein-coding, 30 tRNA, and 4 rRNA genes. Phylogenetic analysis well resolved that *O. nuda* clustered with *O. xylocarpa* and *O. emarginata*. The plastome of *O. nuda* will provide informative genomic resources for further phylogenetic studies.

*Ormosia nuda* (F. C. How) R. H. Chang and Q. W. Yao is classified in the subfamily Papilionoideae of the legume family (Fabaceae). It is evergreen tree with a distribution in east and south China. As a synonym of *O. nuda*, *O. yaanensis* N. Chao was once published as a new species in 1985 with a single existing tree in Yaan City of Sichuan Province (Chao 1985). *Ormosia* comprises approximately 130 species, however, its taxonomy and evolutionary history remain unclear due to the lack of a robust species-level phylogenetic analyses (Liu et al. 2019). Plastome have been shown to resolve phylogenetic relationships at different taxonomic levels. Therefore, the plastome of the specimen of *O. nuda* from Yaan was assembled and analyzed to provide more molecular loci for further phylogenetic and evolutionary studies of *Ormosia*.

Plant material was collected from Yanjia Mountain in Yaan City of Sichuan province (30°6′38.33″N, 103°2′53.81″E), and the voucher specimen (no. WYH2020001) was deposited in the herbarium of Southwest University (SWCTU Qian Wang, wangqian123@swu.edu.cn). The total genomic DNA was extracted from silica gel-dried leaves. A 150-bp paired-end library was generated and sequenced on Illumina HiSeq 4000 platform at the Beijing Novogene Bioinformatics Technology Co., Ltd. (Nanjing, China). About 2 GB raw reads were obtained and used to assemble the plastome with GetOrganelle (Jin et al. 2020). Two small gaps within AT-riched intergenic spacers were failed to sequencing even by polymerase chain reaction. A reference-based annotation was executed in Geneious Prime (v. 2020.0.5, Biomatters Ltd.) with manual adjustment. The raw reads and plastome sequence were deposited in SRA and GenBank, respectively.

The plastome of *Ormosia nuda* was 173,789 bp in length with a typical quadripartite structure, containing two inverted repeat (IR) regions of 40,599 bp separated by a large single-copy (LSC) region of 73,847 bp and a small single-copy (SSC) region of 18,744 bp. The plastome GC content was 35.8%. It was comprised 111 unique genes including 77 protein-coding genes (PCGs), 30 tRNAs, and 4 rRNAs. Thirty genes were duplicated by IRs. The plastome of *O. nuda* was well collinear and of similar length by comparing with other published *Ormosia*.

To determine the phylogenetic location of *O. nuda* within *Ormosia*, PCGs and noncoding regions (NCRs, including introns and intergeneric regions) of *O. nuda* and six published *Ormosia* were analyzed. Eight legume species (Figure 1) were designated as outgroups. Each PCG and NCR were aligned, respectively, using the Mafft Multiple Alignment V.1.4.0 plugin (Biomatters Ltd.) implemented in Geneious Prime. Ambiguously aligned sites in all these alignments were removed using GBLOCKS v.0.91b (Castresana 2000; Talavera and Castresana 2007) with default parameters. Alignments of 77 PCGs and 140 NCRs were optimized and then concatenated to final PCG and NCR matrixes. The maximum likelihood (ML) and Bayesian inference (BI) phylogenetic reconstructions were executed by using RAxML V.4.0 plugin (Biomatters Ltd.) implemented in Geneious Prime and MrBayes V.3.2.7a (Ronquist et al. 2012), respectively, under the GTRGAMMA nucleotide substitution model.

**Figure 1.**
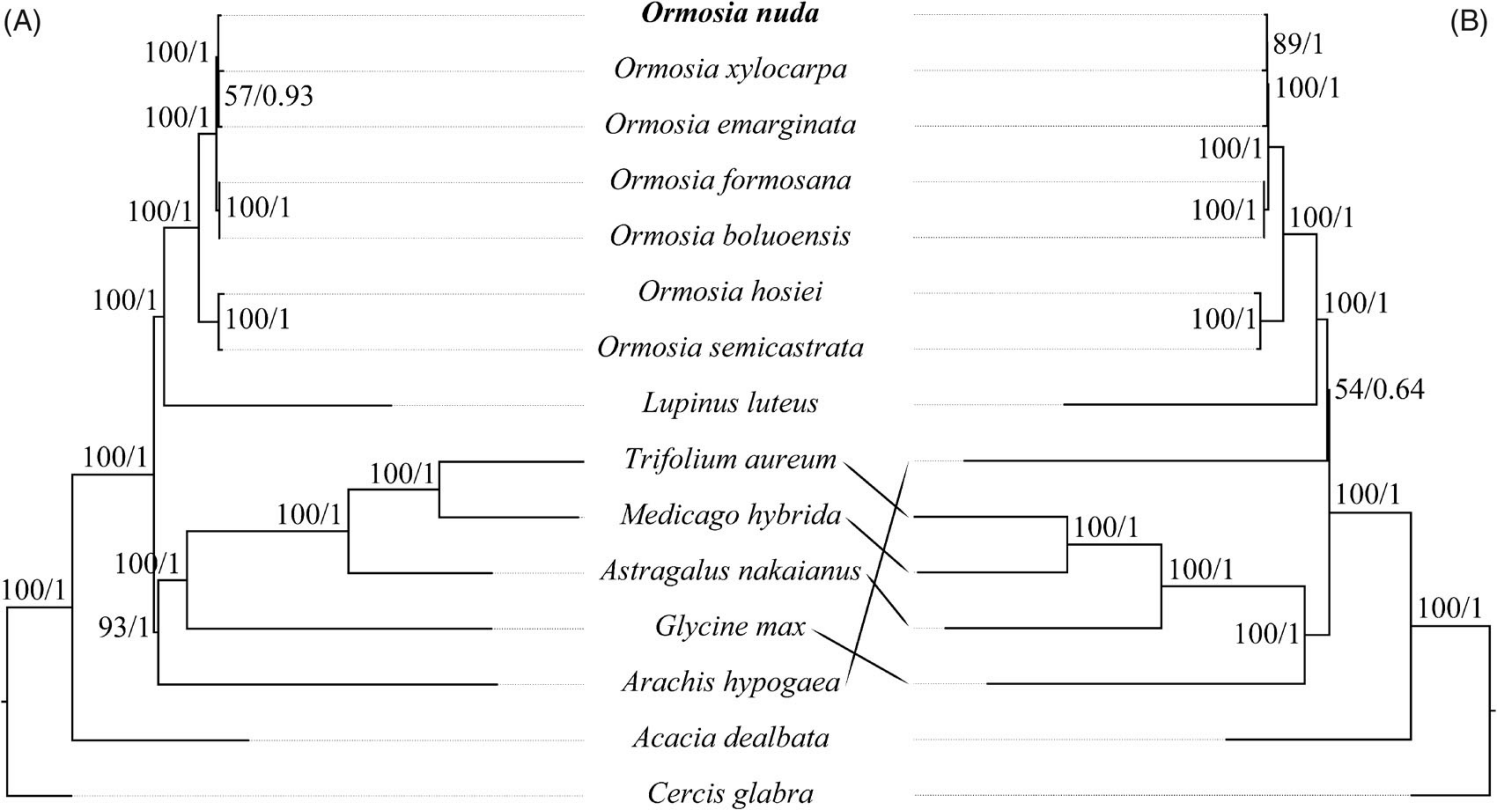
Phylogenetic tree of sampled *Ormosia* species and outgroups based on 77 protein-coding genes (A) and noncoding regions (B) of plastomes. Numbers beside nodes represent maximum likelihood bootstrap percentages/Bayesian inference posterior probabilities. Bold type marks species sequenced in this study. The analyzed species and corresponding GenBank accession number are as follows: *Acacia dealbata* (NC_034985), *Arachis hypogaea* (NC_026676), *Astragalus nakaianus* (NC_028171), *Cercis glabra* (NC_036762), *Glycine max* (NC_007942), *Lupinus luteus* (NC_023090), *Medicago hybrida* (NC_027153), *Ormosia boluoensis* (MN886968), *O. emarginata* (NC_045104), *O. formosana* (MT258921), *O. hosiei* (NC_039418), *O. nuda* (MW450912), *O. semicastrata* (NC_045106), *O. xylocarpa* (NC_045105), and *Trifolium aureum* (NC_024035).

Both ML and BI phylogenetic results fully resolved *O. nuda* in a clade with *O. emarginata* and *O. xylocarpa*, which is sister to the clade (*O. boluoensis* + *O. formosana*) (Figure 1). The five spices form a clade that is sister to the clade comprising the remaining two species, *O. hosiei* and *O. semicastrata*. This is different from the result of Liu et al. (2019), but consistent with that of Guo et al. (2020) and Wang et al. (2020). The phylogenetic tree based on PCGs yields a relationship of *O. nuda* (*O. emarginata*, *O. xylocarpa*) with very low bootstrap support value (BS = 57) and Bayesian posterior probability (PP = 0.93) (Figure 1A). While that based on NCRs supports the relationship of *O. emarginata* (*O. nuda*, *O. xylocarpa*) (BS = 89, PP = 1; Figure 1B). NCRs are more informative on resolving intra-generic relationships of *Ormosia*. These results indicating that plastomes will be beneficial for further systematic, phylogenetic, and genetic diversity of *Ormosia*.

## Data Availability

The genome sequence data that support the findings of this study are openly available in GenBank of NCBI at [https://www.ncbi.nlm.nih.gov] (https://www.ncbi.nlm.nih.gov/nuccore/MW450912.1/) under the accession no. MW450912. The associated BioProject, SRA, and Bio-Sample numbers are PRJNA694335, SRP302991, and SAMN17517253, respectively.
